# Interaction Between microRNAs and Myeloid-Derived Suppressor Cells in Tumor Microenvironment

**DOI:** 10.3389/fimmu.2022.883683

**Published:** 2022-05-11

**Authors:** Lifei Liang, Xiaoqing Xu, Jiawei Li, Cheng Yang

**Affiliations:** ^1^ Department of Urology, Zhongshan Hospital, Fudan University, Shanghai, China; ^2^ Shanghai Key Laboratory of Organ Transplantation, Shanghai, China; ^3^ Fudan Zhangjiang Institute of Fudan University, Shanghai, China

**Keywords:** MDSC, miRNA, tumor microenvironment, tumor resistance, exosomes

## Abstract

Myeloid-derived suppressor cells (MDSCs) are a heterogeneous population of cells generated during a series of pathologic conditions including cancer. MicroRNA (miRNA) has been considered as a regulator in different tumor microenvironments. Recent studies have begun to unravel the crosstalk between miRNAs and MDSCs. The knowledge of the effect of both miRNAs and MDSCs in tumor may improve our understanding of the tumor immune escape and metastasis. The miRNAs target cellular signal pathways to promote or inhibit the function of MDSCs. On the other hand, MDSCs transfer bioinformation through exosomes containing miRNAs. In this review, we summarized and discussed the bidirectional regulation between miRNAs and MDSCs in the tumor microenvironment.

## Introduction

Tumor immune escape and metastasis are critical steps in cancer progression, which have been implicated in the failure of cancer immunotherapies. To achieve that, cancer helper cells in the tumor microenvironment (TME), including regulatory T cells (T-regs), tumor-associated macrophages (TAMs), cancer-associated fibroblasts (CAFs), and myeloid-derived suppressor cells (MDSCs), make a great contribution to protect cancer cells from being recognized and eliminated by the immune system (1).

Among all the immune suppressive cells in TME, MDSCs played a vital role in cancer escape from host immune surveillance (2). MDSCs are a group of immunosuppressive cells differentiated from myeloid cells stimulated by chronic inflammation and other pathological conditions (3). MDSCs were characterized by different phenotypes and functions. In humans, MDSCs were divided into two main groups named monocytic myeloid-derived suppressor cells (M-MDSCs) and polymorphonuclear myeloid-derived suppressor cells (PMN-MDSCs), also referred to as granulocytic myeloid-derived suppressor cells (G-MDSCs) (4). These two groups of MDSCs were defined as CD33^+^CD11b^+^HLA-DR^-/lo^CD14^+^CD15^-^ and CD33^+^CD11b^+^HLA-DR^-/lo^CD14^-^CD15^+^, respectively. In mice, M-MDSCs and G-MDSCs or PMN-MDSCs were defined as CD11b^+^Ly-6G^-^Ly-6C^hi^ and CD11b^+^Ly-6G^-^Ly-6C^lo^ cells (4–6). Recently, some studies defined early-stage myeloid-derived suppressor cells (e-MDSCs) characterized with the phenotype of CD3^-^CD14^-^CD15^-^CD19^-^CD56^-^HLA-DR^-^CD33^+-^ and reported their functions and development (7).

MicroRNA (miRNA) has been investigated in different cancers, and the evidence of its involvement in the regulation of the tumor microenvironment has been of much interest. Some studies found that miRNA expression could be mediated by cancer-derived factors, MDSCs, or through direct miRNA import *via* extracellular vesicles (8). miRNAs have been proven to regulate MDSCs through various ways including disrupting the differentiation of myeloid cells, increasing proliferation, and affecting the immunosuppression and function of immune cells. In the hematopoietic system, microRNAs are treated as important regulators of myeloid lineage induction and differentiation, and recent studies have begun to unravel the crosstalk between miRNAs and MDSCs in TME (9).

Exosomes were first found in 1981 (10) as rubbish carriers to clean degraded or wasted cell components. However, with the deepening of the research, the positive function of exosomes like intracellular communication or immune response was gradually exposed to us (11). Although controversial, thought provoking, studies have revealed that tumor-derived exosomes from MDSCs can carry miRNAs that are parts of the tumor microenvironment and protect tumor cells (9, 12). Furthermore, MDSC-derived exosomes are also delivered to support progression and modulate the expansion and suppressive function of MDSCs themselves (13, 14). MDSC-derived exosomes carrying miRNAs would make MDSCs more convenient to interact with tumor cells. On the other hand, miRNAs transferred by tumor-derived exosomes can make a long-distance travel in body fluid to regulate the expansion and function of MDSCs, which assist tumor angiogenesis and invasion.

To create a suitable microenvironment, tumor cells secrete miRNAs, cytokines, and other molecules to escape from immune surveillance. The expression of miRNAs controls the function of MDSCs and inhibitory immune cells, such as T-regs (15). As an essential component of tumor microenvironment, MDSCs lives in the inflammatory environment, causing tumor progression and helping tumors grow and suppressing immunity as well. MDSCs also regulate miRNAs in the microenvironment. Both MDSC and tumors can regulate miRNA expression to ease their increment and metastasis. Furthermore, the exosomes derived from MDSCs and tumors can transport miRNAs locally and over long distance, so that builds a bridge between MDSCs, tumor cells, and the immune network.

Still, there are challenges remaining. The origin of miRNA is complex and needs further validation, and whether the miRNA secreted by MDSCs or tumor cells influences other immune cells in the microenvironment should be clarified. Solving these questions might help in finding the way blocking miRNAs specifically.

In this review, we focus on the mechanisms of how miRNAs exert an effect on MDSC functions, the intercommunication between miRNAs and MDSCs, their effect on the components of the tumor microenvironment, and progress on miRNAs in the exosomes derived from tumors and MDSCs.

## MDSCs Regulates miRNAs in the Tumor Microenvironment

Several studies have shown that not only miRNAs regulate MDSC function and differentiation, but MDSCs could also modulate miRNA expression to promote cancer invasion and metastasis (16). It was reported that MDSCs marked with the myeloid differentiation factor schlafen4 (SLFN4), a regulator of myeloid cell differentiation, were identified in gastric cancer, especially in the preneoplastic changes infected by *Helicobacter* (17). miR-130b from SLFN4^+^MDSC promoted gastric epithelial cell proliferation and was essential for MDSC expressing the function of T-cell suppression (18). As for papillary thyroid carcinoma (PTC), the PMN-MDSCs showed a great effect on PTC progression. It decreased the expression of miR-486-3p, which targeted the NF-kB pathway directly and thus activated the NF-kB pathway and facilitated PTC invasion and, in turn, increased PMN-MDSC expansion and function of repressing T cells (15). However, the basic mechanism or the key cytokines regulating this axis still need to be further studied.

The progression of ovarian carcinoma was investigated to be highly correlated with MDSCs and cancer stem cells (CSCs), which are dispensable for cancer advancement in TME. MDSCs upregulated miR-101 expression and further repressed C-terminal binding protein-2 (CtBP2), a corepressor gene targeting stem cell core genes directly, and thus promoted the stemness and invasion of cancer cells. Thus, the MDSCs-miR-101-CtBP2-cancer cell core genes axis was therefore considered as a potential target for antitumor immunotherapy (19).

## MDSCs-Derived Exosomal miRNAs Mediate Tumor Progression

Studies have shown that not only tumor-derived exosomes or extracellular vehicles can mediate the expansion and suppressive function of MDSCs by delivering miRNAs, but MDSC-derived exosomes can also carry miRNAs, which have been certified using next-generation sequencing (13) and exert influence on tumor invasion and metastasis (14).

miR-143-3p in G-MDSC-derived exosomes inhibited integral membrane protein 2B (ITM2B) and activated the PI3K/AKT pathway, thus promoting the cell proliferation of lung cancer (20). It was reported that MDSCs were involved in the resistance of chemotherapy for breast cancer and identified its underlying mechanism with doxorubicin-induced MDSCs (21). The DOX-MDSC produced exosomal miR-126a and promoted the induction of IL-13^+^Th2 T cells, which secreted IL-13 to increase the proliferation of DOX-MDSC and exosomal miR-126a. The study also found that the exosomal miR-126a of DOX-MDSC repressed MDSC apoptosis and contributed to tumor angiogenesis in an S100A8/A9-dependent way (22).

Geis-Asteggiante et al. provided evidence that MDSC-derived exosomes carry miRNAs. Four differentially abundant miRNAs (miR-7022, miR-7062, miR-5134, and miR-704) had predicted mRNA targets that were part of the apoptotic pathway-inducing Fas, which was also a validated target of miRNA-98a (14). Another 4 miRNAs in MDSC-derived exosomes included miR-9, miR-494, miR-233, and miR-690, which were capable of affecting the cell cycle, resulting in suppressing the differentiation of myeloid cells and increasing MDSC proliferation (23, 24). miR-155, a key miRNA enriched in MDSC-derived exosomes, increases IL-10 production in MDSC and contributes to the crosstalk between MDSCs and macrophages (25–27). miR-155 mediates the MDSC function of suppression through at least two ways including SOCS1 and inhibiting the generation of CD4^+^Foxp3^+^ regulatory T cells (28).

## The miRNAs in the Tumor Microenvironment Regulate MDSCs Function by Different Signal Pathways

### JAK/STAT Pathway

The Janus kinase/signal transducers and activators for the transcription (JAK/STAT) pathway show great influence on cell proliferation, differentiation, and inducing inflammatory microenvironment for cancer. The STAT family is composed of seven members including STATs 1, 2, 3, 4, 5a, 5b, and 6 (29). Among all these proteins, STAT3 seems to be a key protein for the creation of cancer microenvironment and be involved in MDSC development modulated by miRNAs (29–31). miRNAs have been proven to interact with MDSCs, and STAT3 could be a crucial target within it. miR-17-5p and miR-20a downregulated the suppressive function of MDSCs by targeting the 3’UTR of STAT3 to block its expression, which remarkably reduced the production of reactive oxygen species (ROS) and H_2_O_2_ (32). However, only G-MDSCs could be inhibited by miR-17-5p, and miR-20a and M-MDSCs showed less affection. It was also demonstrated that miR-17-5p and miR-20a were regulated by tumor-associated factors and the transfection of these miRNAs could be a possible treatment for tumor immunotherapy. miR-6991-3p was markedly reduced in MDSCs from the tumor microenvironment, which means that miR-6991-3P repressed the MDSC expansion and function of inhibiting T-cell proliferation. STAT3 was proved to be the direct target of mir-66991-3p (33). On the contrary, miR-155 and miR-21 synergistically upregulated STAT3 expression indirectly by targeting SHIP-1 and PTEN, respectively, and eventually enhanced the function and expansion of MDSCs. Both were identified as early indicators for predicting patients’ reactions to glucocorticoid treatment. Both monocytic and granulocytic MDSCs were influenced by the upregulation of miR-155 and miR-21 (25). Studies also revealed that tumor environment-associated factors activate STAT3 and C/EBPb to increase the transcription of miR-21a, miR-21b, and miR-181b (34). Increased levels of these miRNAs disrupted the mixed-lineage leukemia (MII1)-complex and allowed the PMN-MDSCs to exert their immunosuppressive function. The STAT3/CEBPb-miR-21a/b/181b-MII1 axis provided an effective immunotherapeutic manner against cancer. The M-MDSC in the colorectal cancer (CRC) microenvironment secreted CCL17. This chemokine was combined with CR2 and activated the JAK/STAT3 pathway, which awakened the dormant cancer cells and promoted cancer progression clinically (35). miR-124-3p was demonstrated to inhibit the PD-L1 pathway and STAT3 signaling in CRC, which might indicate that miR-124-3p mediated the MDSCs of CRC through the PD-L1/STAT3 pathway (36). This might be a potential therapeutic target to prevent MDSC accumulation and CRC recurrence and metastasis.

For other STAT proteins, STAT6 is found to strengthen the expansion of G-MDSCs while it weakens the expansion of M-MDSCs, and STAT6 could be inhibited by the overexpression of miR-449c and increases the accumulation of M-MDSCs (37).

### SOCS Signal

Suppressor cytokine signaling (SOCS)1, a member of the SOCS family, is an inhibitor of the JAK/STAT pathway (38), which mediates the expansion and suppressive function of MDSCs. A recent study reported that the expression of miR-155 was required for the suppressive function of MDSCs and was a necessity for the T-reg induced by MDSCs (28). miR-155 mediated MDSCs by targeting SOCS1 directly and eliminated the inhibition of the JAK/STAT pathway conducted by SOCS1, thus contributing to the accumulation of MDSCs and exerting immunosuppressive function.

It is known that SOCS3 negatively mediates the expansion and function of MDSCs *via* inhibiting STAT3 (39). miR-30a was demonstrated to target SOSC3 directly and increased the activation of STAT3, participated in MDSC proliferation and immunosuppression by inducing Arg-1, IL-10, and ROS, thus eventually resulting in B lymphoma deteriorated with upregulating MDSC infiltration and suppression (40). miR-9 was also identified as activating the JAK/STAT pathway *via* targeting SOCS3 and promoted the development of eMDSCs in breast cancer. miR-9 improved and coordinated with miR-181a expression, which was also an inhibitor of the STAT pathway by bounding to PIAS3 (41).

However, in ovarian cancer, miR-101 was reduced while SOCS2 gene expression increased. The transection of miR-101 could remarkably downregulate SOCS2 and thus inhibit the invasion and metastasis of ovarian cancer cells (42).

### PTEN and PI3K/Akt Pathway

It is well known that PTEN is a key regulator in neutrophils’ spontaneous death (43) and the downregulation of CXCR4-mediated chemotaxis (44). miR-494, induced by tumor-derived factors, such as TGF-β1, is reported as an activator of MDSCs. miR-494 downregulates PTEN and activates the PI3K/Akt pathway to enhance the MDSCs’ chemotaxis mediated by CXCR4 and change the normal progress on apoptosis and cell death, which promotes the accumulation of MDSCs in tumors (45). The activation of the Akt pathway also facilitates tumor invasion and metastasis. Studies also found that miR-200c, induced by GM-CSF, showed a positive effect on the proliferation and suppressive function of MDSCs by targeting PTEN and friend of Gata2 (FOG2) and further activated the PI3K/Akt and STAT3 pathways (24). miR-21 is demonstrated to regulate MDSC expansion by targeting PTEN, which increases the activity of the STAT3 pathway (25).

### RUNX1/YAP Pathway

The classical myeloid differentiation-related gene runt-related transcription factor 1 (Runx1) is modulated during the differentiation and maturation of MDSCs. RUNX1 is one of the core-binding family transcriptional factors and is essential to hematopoietic lineage and myeloid expression and differentiation (46, 47). Recently, miR-9 has been demonstrated to be inversely correlated with the expression of RUNX1 in lung cancer and miR-9 would inhibit MDSC differentiation and aggravate the suppressive function of MDSCs. Direct injection of miR-9 successfully repressed tumor development. However, further clinical studies were needed to verify whether the miR-9 inhibitor was an effective anti-tumor immunotherapy (46). It was also found that miR-21 maintains the accumulation of MDSCs in the microenvironment of lung cancer *via* inhibiting the expression of RUNX1 (48). In addition, RUNX1 was found to downregulate the expression of yes-associated protein (YAP) to deteriorate tumor progression (49). Thus, the miR-21/RUNX1/YAP axis could be another underlying mechanism for miR-21 mediating MDSCs and tumor growth.

### Targeting CCAAT Enhancer-Binding Protein

CCAAT enhancer-binding protein (CEBP) transcription factors show a significant effect on the proliferation and differentiation of myeloid cells (50). miR-486 was considered as a regulator of myeloid cell differentiation and apoptosis by targeting CEBPα, and the expression between miR-486 and CEBPα was inversely correlated in tumor-induced M-MDSCs (TM-MDSCs). TM-MDSCs are a group of cells involved in tumor angiogenesis and immunity escape by suppressing the function of T cells. However, either miR-486 or CEBPα overexpression would inhibit the differentiation of myeloid cells, indicating that both miR-486 and CEBPα were involved in the expansion of TM-MDSCs in tumors (51). Based on the suppressive function of MDSCs in tumor-bearing mice, △^9^-tetrahydrocannabinol (THC)-induced MDSCs were used to confirm that miR-690 had great potential on maintaining the immunosuppression of MDSCs *via* decreasing the expression of CEBPα and decaying their terminal differentiation (23). Although some studies utilized miR-155 as a promoter for the induction of MDSCs in tumors and the lack of miR-155 led to the deterioration of solid tumor (52), Kim et al. found that miR-155 negatively correlated with the expression of MDSCs and identified CEBP as a target of the miR-155-mediating recruitment of MDSCs (53).

### Other Targets

Hypoxia-inducible factor 1α (HIF-1α) plays a major role in converting MDSC differentiation and function in the tumor microenvironment with hypoxia (54). Under hypoxia, miR-210, elevated by HIF-1α, affected Arg1, IL-16, and CXCL12 expression and further exacerbated the function of MDSCs, promoting the development of tumors (55). HIF-1α, a direct target of miR-155, was upregulated in miR-155-deficient MDSCs, which increased the expression of chemokines and further accelerated MDSC infiltration in TME (56). Other miRNAs also presented the function of tumor-inhibiting, for instance, miR-233 remarkably slowed the progression of the tumor by repressing myeloid cell differentiation to MDSCs *via* targeting myocyte enhancer factor 2C (MEF2C) (57).

miR-34a contributes to the expansion of MDSCs by suppressing the expression of N-myc. Instead of promoting MDSC proliferation, miR-34a reduces the apoptosis of MDSCs without an effect on progenitor cell differentiation to increase their infiltration (58). miR-34a was also demonstrated to be the driver of MUC1, promoting C-Myc expression in AML-related EVs and the expansion of MDSCs (59). Moreover, miR-34 was confirmed to have a synergistic effect on MDSCs with TWIST (60), a transcription factor of the bHLH family, and contributes to cancer progression and immune resistance (61).

It was elaborated that the PEG2/miR-10a/AMPK axis played an undeniable role in chemotherapy-resistant breast cancer. The PEG2 released by doxorubicin-resistant cancer cells stimulated miR-10a expression, which was the activator of the AMPK pathway, thus leading to the upregulation of MDSC immunosuppression (62). Further studies of this axis would provide a silver lining for treating chemotherapy-resistant tumors.

It is known that CXCR4 plays an essential role in recruiting MDSCs and promoting the progression and metastasis of CRC (63). miR-133a-3p was proven to be involved in this process by activating RhoA/ROCK signal and was mediated by lncRNA XIST (64).

Zhao et al. came up with a prognostic model of 4-circulating miRNAs (miR-21, miR-130b, miR-155, and miR-28) to predict the outcome of diffuse large B-cell lymphoma and tested its validity with a cohort study. They also revealed the association between the 4-circulating miRNA model and the RAS signal pathway and how the tumor environment affects lymphoma. In tumor progression, the alteration of these miRNAs led to RAS pathway activation and MDSC upregulation (65).

### Tumor-Derived Exosomes and Extracellular Vesicles

**Figure 1:**
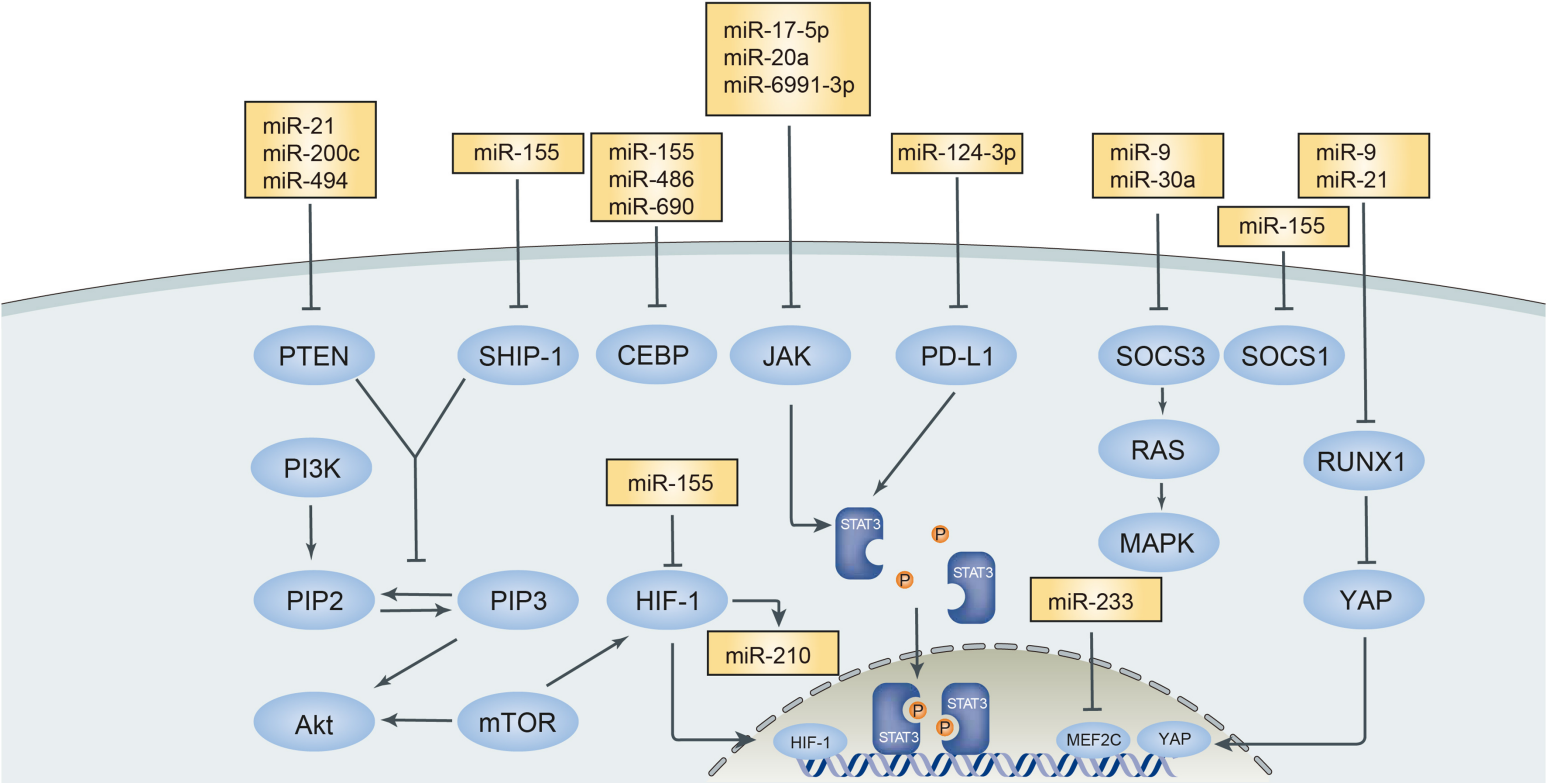
Main signal pathways interacted with microRNAs in tumor microenvironment. MicroRNAs in tumor microenvironment exert positive or negative effect on MDSCs targeting different signal pathways. PTEN, Phosphatase and tensin homolog; SHIP-1, Src Homology 2-containing inositol phosphatase-1; CEBP, CCAAT/enhancer binding protein; JAK-STAT3, Janus kinase-signal transducer and activator of transcription; PD-L1, Programmed death-ligand 1; SOCS3, suppressor cytokine signaling 3; RUNX1, runt-related transcription factor 1; MEF2C, MADS box transcription enhancer factor 2, polypeptide C.

Exosomes and extracellular vehicles (EVs) can carry and deliver miRNAs to MDSCs and contribute to the regulation of MDSCs as miRNAs secreted *in situ*. Tumors produce EVs and exosomes as a manner of augmenting the immunosuppression of MDSCs in the tumor microenvironment and assisting their invasion and escape from surveillance of immune cells (66, 67). miR-9 and miR-181a in exosomes derived from breast cancer were identified to target SOCS3 and PIAS3, respectively, and further activated the JAK/STAT pathway, thus promoting the amplification and development of eMDSC (41). The miR-21a in exosomes from Lewis lung carcinoma cells accelerates tumor growth through targeting programmed cell death protein 4 (PDCD4) through activating the autocrine production of IL-6 and phosphorylation of the STAT3 signaling pathway and thus enhances the expansion of MDSCs and tumor growth (68). Furthermore, miR-21 in oral squamous cell carcinoma (OSCC) enhanced the immunosuppressive function of MDSCs through an miR-21/PTEN/PD-L1 axis (69) and in esophageal squamous cell carcinoma (ESCC), miR-21 activated the STAT3 pathway carried by cancer-associated fibroblast (CAF)-secreting exosomes, which upregulated the induction of M-MDSC corporate with IL-6 (70). Has-miR-494-3p and has-miR-1260a in pancreatic ductal adenocarcinoma (PDAC)-derived exosomes mediated the suppressive function of MDSCs in an Smad4-dependent way (71). miR-10a and miR-21a carried by hypoxia-stimulated glioma-derived exosomes (H-GDEs) showed a more aggressive mediating MDSC suppression on CD8^+^T cells than N-GNEs did. Both miRNAs in exosomes regulated MDSCs separately through miR-10a/Rora/IκBα/NF-κB and miR-21/PTEN/PI3K/AKT pathways (72). The transfer of miR-29a and miR-92a showed similar effects like miR-10a and miR-21a in glioma with the hypoxia tumor environment. Hypoxia-induced glioma produced exosomes to carry miR-29a and miR-92a and transferred them to promote the differentiation of functional MDSCs (73). MiR-107 in the gastric cancer-derived exosomes was caught by MDSCs and inhibited the expression of *DICER1* and *PTEN* genes, thus increasing the expansion of MDSCs and ARG1 expression, respectively (74). miR-1246 in glioma-derived exosomes was demonstrated to mediate MDSC differentiation and activation in a dual-specificity phosphatase 3 (DUSP3)/extracellular signal‐regulated kinase (ERK)-dependent mechanism. The expression of exosomal miR-1246 was correlated with glioma recurrence (75). The main signal pathways of MDSCs that interacted with microRNAs in the tumor microenvironment are illustrated in **Figure 1**.

Tumor-derived extracellular vesicles serve as a communication tool for the crosstalk between cells by carrying proteins, RNAs, and DNAs (76). EV-carried miRNAs could mediate the expansion and suppressive function of MDSCs *via* targeting different points or pathways in the tumor microenvironment (77, 78). CLL-derived EVs contributed to MDSC accumulation by transferring miR-155 and could be inhibited by vitamin D (79). A line of miRNAs (miR-146a, miR-155, miR-125b, miR-100, miR-125a, let-7e, miR-146b, miR-99b) in the EVs derived from melanoma was associated with the accumulation of MDSCs and the immunotherapy of checkpoint inhibitors (67).

## Perspective

Although MDSCs have been studied for decades, the bidirectional regulation between microRNA and MDSCs still needs further investigation. The first question is where the miRNAs are from. MicroRNA can be secreted by various cells, including MDSCs themselves. The origin of miRNA is complex and needs further validation. The next question is whether the miRNA secreted by MDSCs influences other immune cells in the microenvironment. Immune regulation is a network, regulated by cytokines, miRNAs, and other molecules. It is well known that MDSCs and cancer cells secrete exosomes, which contain many miRNAs, and regulate other immune cell functions. Catherine Fenselau et al. used next-generation sequencing, identifying more than 1,400 miRNAs in MDSC-derived exosomes, and 24% of them were related to MDSC (13). Therefore, using advanced technologies, such as the third-generation sequencing, will help us investigate more information about miRNAs in exosomes. In the future, targeting specific miRNA could block or enhance MDSC function. Through systemic or carrier-loaded delivery, it might regulate MDSC function using miRNA-based drugs.

## Conclusion

Immune escape and chemotherapy resistance are tough problems for the treatment of tumors. However, with continuous studies of factors in the tumor microenvironment, great progress has been made on miRNAs and MDSCs. Multiple studies have elaborated that miRNAs mediate MDSC expansion and function *via* targeting pathways or transcriptional factors including STAT, PTEN, RUNX1, SOCS, CEBP, and other target points. It was also described that MDSCs regulated miRNA expression to facilitate their proliferation and create favorable conditions for tumor growth and invasion. Other than the mechanisms of direct interaction between miRNAs and MDSCs, studies tried to figure out if there were some indirect ways to achieve the same outcome as their counterparts did. The exosomes and extra vehicles secreted from cancer cells and MDSCs carried miRNAs and made a difference in the tumor microenvironment. However, more studies are needed to verify the accuracy and feasibility of the results and data existing.

## Author Contributions

LL and JL drafted the manuscript. XX and CY revised the manuscript. CY and JL conceived the review design. All authors contributed to the article and approved the submitted version.

## Funding

This study was supported by National Natural Science Foundation of China (82170765 to CY), National Key R&D Program of China (2018YFA0107502 to CY), Shanghai Rising-Star Program (19QA1406300 to CY), and 2019 Shanghai Youth Talent Development Program (to CY).

## Conflict of Interest

The authors declare that the research was conducted in the absence of any commercial or financial relationships that could be construed as a potential conflict of interest.

## Publisher’s Note

All claims expressed in this article are solely those of the authors and do not necessarily represent those of their affiliated organizations, or those of the publisher, the editors and the reviewers. Any product that may be evaluated in this article, or claim that may be made by its manufacturer, is not guaranteed or endorsed by the publisher.
